# Paracrine interactions between primary human macrophages and human fibroblasts enhance murine mammary gland humanization *in vivo*

**DOI:** 10.1186/bcr3215

**Published:** 2012-06-25

**Authors:** Jodie M Fleming, Tyler C Miller, Michal Kidacki, Erika Ginsburg, Christina H Stuelten, Delisha A Stewart, Melissa A Troester, Barbara K Vonderhaar

**Affiliations:** 1 Mammary Biology and Tumorigenesis Laboratory, Center for Cancer Research, National Cancer Institute, Bethesda, MD 20892, USA; 2 North Carolina Central University, Department of Biology, Durham, NC 27707, USA; 3 Laboratory of Cell and Molecular Biology, Center for Cancer Research, National Cancer Institute, Bethesda, MD 20892, USA; 4 Department of Epidemiology, Gillings School of Public Health. University of North Carolina at Chapel Hill, Chapel Hill, NC 27599, USA

## Abstract

**Introduction:**

Macrophages comprise an essential component of the mammary microenvironment necessary for normal gland development. However, there is no viable *in vivo *model to study their role in normal human breast function. We hypothesized that adding primary human macrophages to the murine mammary gland would enhance and provide a novel approach to examine immune-stromal cell interactions during the humanization process.

**Methods:**

Primary human macrophages, in the presence or absence of ectopic estrogen stimulation, were used to humanize mouse mammary glands. Mechanisms of enhanced humanization were identified by cytokine/chemokine ELISAs, zymography, western analysis, invasion and proliferation assays; results were confirmed with immunohistological analysis.

**Results:**

The combined treatment of macrophages and estrogen stimulation significantly enhanced the percentage of the total gland humanized and the engraftment/outgrowth success rate. Timecourse analysis revealed the disappearance of the human macrophages by two weeks post-injection, suggesting that the improved overall growth and invasiveness of the fibroblasts provided a larger stromal bed for epithelial cell proliferation and structure formation. Confirming their promotion of fibroblasts humanization, estrogen-stimulated macrophages significantly enhanced fibroblast proliferation and invasion *in vitro*, as well as significantly increased proliferating cell nuclear antigen (PCNA) positive cells in humanized glands. Cytokine/chemokine ELISAs, zymography and western analyses identified TNFα and MMP9 as potential mechanisms by which estrogen-stimulated macrophages enhanced humanization. Specific inhibitors to TNFα and MMP9 validated the effects of these molecules on fibroblast behavior *in vitro*, as well as by immunohistochemical analysis of humanized glands for human-specific MMP9 expression. Lastly, glands humanized with macrophages had enhanced engraftment and tumor growth compared to glands humanized with fibroblasts alone.

**Conclusions:**

Herein, we demonstrate intricate immune and stromal cell paracrine interactions in a humanized *in vivo *model system. We confirmed our *in vivo *results with *in vitro *analyses, highlighting the value of this model to interchangeably substantiate *in vitro *and *in vivo *results. It is critical to understand the signaling networks that drive paracrine cell interactions, for tumor cells exploit these signaling mechanisms to support their growth and invasive properties. This report presents a dynamic *in vivo *model to study primary human immune/fibroblast/epithelial interactions and to advance our knowledge of the stromal-derived signals that promote tumorigenesis.

## Introduction

Mammary gland development and function depends on intricate interactions of the functional epithelial cells with local stromal cells, including fibroblasts, adipose, endothelium, immune and nerve cells [1-4]. The mammary gland is a particularly unique organ as the majority of its development occurs postnatally. Systemic and locally produced steroids and growth factors orchestrate the outgrowth and differentiation of the epithelium throughout the gland during puberty, followed by additional rounds of proliferation and differentiation during pregnancy, lactation, and involution [1,3]. Furthermore, analyses of breast tissue from human autopsy and surgical specimens suggest that additional morphologic changes in the epithelium repeatedly occur during each menstrual cycle [5]. These morphological changes are a result of hormone- and growth factor-stimulated alterations in proliferation, apoptosis, protein expression, and cell kinetics. It is critical to understand the signaling networks that drive these cyclic changes, for many of the signaling mechanisms that control them are often exploited by tumor cells to support their growth and invasive properties.

Much of our knowledge about these processes in the human breast has been extrapolated from mouse and rat models. Attempts to recapitulate human breast morphogenesis in the mouse mammary gland using direct injections or collagen embedded cells failed to support proliferation of breast epithelial cells in mouse models. However, injection of human breast fibroblasts into the cleared mammary fat pad prior to injection of human breast epithelial cells (xenograft humanized mammary gland model) [6] stimulates human epithelial cell proliferation and promotes the organization of differentiated acini structures, and leads to successful engraftment of mouse mammary fat pads with human breast epithelial cells. This innovative model allows the investigator to observe fibroblast-epithelial interactions, with either normal or cancer-derived cells, in an orthotopic *in vivo *model.

In addition to fibroblasts, the homeostasis of breast epithelial cells depends upon interactions with many different types of stromal cells. In particular, macrophages comprise a vital, functional component of the mammary microenvironment and are essential for normal mammary gland development [7-10]. During development, macrophages are recruited and localized in abundance to the neck of the developing terminal end buds. Their removal during postnatal development by either genetic manipulation or gamma irradiation results in reduced formation of terminal end buds and restricted outgrowth and branching of the epithelial ductal tree [9,11]. Although a plethora of data suggests a central role for macrophages during mammary development, the precise mechanisms remain unknown. It is hypothesized that they promote epithelial growth throughout the gland by supplying growth factors, proteases, cytokines and angiogenic factors [12].

Macrophages play a role not only in normal mammary development but also in breast cancer development, progression, and metastasis. Consistent with cellular alterations that occur during tumorigenesis, tumor-associated macrophages (TAMs) display distinct phenotypes and behavior compared to those in non-malignant tissue [13]. TAMs secrete proteases that facilitate degradation of basement membrane and extracellular matrix for facilitation of tumor invasion and metastasis. Additionally, TAMs supply epidermal growth factor (EGF) and Wnt ligands that promote cell growth and vascular remodeling, respectively [14-16]. Clinical data support these observations; for example, a high density of TAMs is correlated with poor prognosis and decreased survival in breast cancer patients [16-18].

Given the fundamental role macrophages perform in mammary development and function, as well as during tumorigenesis, we hypothesized that the introduction of human macrophages would enhance humanization of the murine mammary gland and permit an intimate first look at human immune and stromal-derived paracrine cell interactions in a dynamic *in vivo *model. Herein, we demonstrate that primary human peripheral blood macrophages enhance humanization by enhancing fibroblast proliferation and engraftment of the mammary fat pad, thereby providing a larger stromal bed for breast epithelial growth and acini formation. We have identified two specific paracrine mechanisms involved in enhancement of humanization: increased fibroblast proliferation stimulated by macrophage-derived TNFα as well as a macrophage-stimulated increase in matrix metalloproteinase (MMP)-9 expression and proteinase activity. Collectively, our data demonstrate intricate mechanisms of primary human immune and stromal-derived paracrine interactions in a humanized xenograft model of the mammary gland.

## Materials and methods

### Collection and processing of patient samples and cell culture

Collection of patient samples was performed in accordance with the guidelines of the National Cancer Institute's Institutional Review Board, under four separate approved protocols OH99-C-NO57, 02-C-0077E, 04-C-0199, OHSR4789, and 99-CC-0168. Written informed consent was obtained from all human subjects as stipulated by the protocols. Breast tissue was collected from premenopausal fasting patients undergoing reduction mammoplasty. The tissue obtained for analyses was considered pathological medical waste; thus, any clinical details of the women, apart from age and race, were unattainable. A pathologist confirmed that each sample was free of malignant or hyperplasic growth. Immediately after surgery a portion of tissue was used for epithelial cell and organoid isolation as described [19]; an additional separate piece of tissue was used for isolation of primary human breast fibroblasts, and the remainder was snap frozen and stored at -80°C. Fibroblasts were isolated by placing < 5 mm pieces of tissue on a scratched cell culture dish, and with time, the fibroblasts crawled out of the tissue to form a monolayer on the dish. The fragments of tissue were removed and the remaining fibroblasts were passaged and plated as monolayer cultures, to expand and ensure fibroblast purity. When necessary, epithelial cells were separated from the stromal cells by differential trypsinization and selective pressure with fibroblast growth medium, DMEM containing 10% FCS (Invitrogen, Gaithersburg, MD, USA). Fibroblasts were grown for a maximum of three passages prior to analysis. Human telomerase reverse transcriptase (hTERT)-immortalized breast fibroblasts were a kind gift from Charlotte Kuperwasser (Tufts University, Boston, MA, USA).

Peripheral blood monocytes and macrophages were collected from premenopausal women undergoing apheresis. Monocytes and macrophages were separated from other cells using Ficoll-Hypaque (Sigma, St. Louis, MO, USA) gradient separation and selection by adherence to tissue culture plastic. Cells were grown in RPMI containing 5% human serum (Invitrogen) for 24 hr then changed to RPMI containing 5% FBS until differentiation. Differentiation was performed via treatment with 20 ng/ml of IFNγ and lipopolysaccharide (LPS, Sigma) in 2% charcoal-stripped serum (Hyclone, Logan, UT, USA) for 48 hr.

T47D breast cancer cells were obtained from American Type Tissue Culture Collection (Manassas, VA, USA) and maintained in RPMI1640 (Invitrogen, Gaithersburg, MD, USA) supplemented with 5% heat-inactivated FBS (Invitrogen), 10 μg/ml bovine insulin (Sigma, St. Louis, MO, USA), and 100 units/ml penicillin-streptomycin (Invitrogen). All cells were maintained at 37°C in a humidified atmosphere with 5% CO_2_. Cells were passaged using trypsinization (0.05% trypsin- ethylenediaminetetraacetic acid (EDTA), Invitrogen) and counted on a hemocytometer using trypan blue exclusion.

### Humanization models

Animal experiments were conducted in accord with accepted standards of humane animal care and approved by the Animal Care and Use Committee at the National Institutes of Health, USA. Female, 3-week-old NOD/SCID mice were randomized into groups with a minimum of five mice per group (APA, Frederick, MD, USA). Mice were anesthetized using inhaled isoflurane (1.0 to 2.5%) vaporized in oxygen, with an intraperitoneal injection of analgesic (Sensorcaine) prior to surgically exposing the abdominal mammary fat pad for injection. Both abdominal mammary glands were humanized as previously described [19] with the following additional steps performed where indicated: mice were supplemented with estrogen via a subcutaneous pellet (0.72 mg β-estradiol, 90-day release; Innovative Research of America, Sarasota, FL, USA) at the time of initial fibroblast injection. Primary human macrophages (7.5 × 10^5^) were injected during both the initial fibroblast injection and second injection of fibroblasts along with primary breast epithelial cells/organoids. Mice were euthanized two months after final injection; glands were removed and immediately imaged and/or fixed in 10% neutral buffered formalin, paraffin-embedded and sectioned. For experiments evaluating fibroblast outgrowths in the presence or absence of macrophages, the same steps were performed as described above without the addition of primary breast epithelial cells/organoids; glands were harvested 10 days after final injection and tissues were processed as described.

### Tumor formation assays

The right abdominal mammary glands were humanized +/- macrophages and the mice were supplemented with estrogen via a subcutaneous pellet, as described above (five mice were injected for each treatment group). Primary human macrophages (7.5 × 10^5^) were injected during both the initial fibroblast injection and second injection of fibroblasts along with 1 × 10^6 ^T47D breast cancer cells. Tumor growth was measured using calipers on a weekly basis. Final tumor volume was calculated ((0.5 × L) × (0.5 × W) × (0.5 × H) × (4/3) × (Π)).

### Comparative PCR expression analysis of differentiated macrophages by colony stimulating factor-1 (CSF-1) or LPS and INFγ

Peripheral blood monocytes were collected from premenopausal women undergoing apheresis and isolated as described above. Differentiation was performed via treatment with 20 ng/ml of IFNγ and LPS or 50 ng/ml of CSF-1 (Peprotech, Rocky Hill, NJ, USA) in 2% charcoal-stripped serum for 5 days. RNA was isolated using the RNeasy kit (Qiagen, Valencia, CA, USA). RNA purity and integrity were confirmed by Agilent 2100 Bioanalyzer. RNA samples (100 ng/sample) were converted into cDNA (Qiagen) and plated on custom-designed PCR arrays (SA Biosciences) containing 43 macrophage differentiation and polarization genes [20] to determine the concordance of macrophage differentiation between the two treatment protocols. Real-time quantitative PCR analysis was performed according to the manufacturer's protocol using an Applied Biosystems 7900HT Fast PCR machine. PCR thresholds were identically adjusted then raw cycle threshold (CT) values were uploaded into the SA Biosciences webpage for comparative analysis, where the CSF-1-treated cells were designated as the reference control. The *P*-values for the averaged relative gene expression between the glyceraldehyde 3-phosphate dehydrogenase (GAPDH)-normalized CSF-1 versus LPS and INFγ treated cells were retrieved and can been seen in Additional File 1, Table S1 with the average change in cycle threshold (ΔCT) values. A two-tailed *t*-test confirmed no significantly differential expression between the two treatment groups across all genes (*P *= 0.7275). Only four genes were significantly differentially expressed, based on *P*-values (*BIRC3*, *CCR7*, *CD163*, and *PTX3*).

### Fluorescence *in situ *hybridization analysis (FISH)

#### Antigen retrieval

Slides were de-paraffinized by three treatments in xylene and then dehydrated in 100% ethanol (Sigma). Antigen retrieval (pepsin) was performed using the Histology Kit (Dako, K5599; Carpinteria, CA, USA) according to the manufacturer's instructions, followed by two washes with 2× SSC (saline-sodium citrate buffer; 0.3 M NaCl, 30 mM trisodium citrate, pH 7.0)). Specimens were then dehydrated in an ethanol series and allowed to dry.

#### Preparation of chromosome paint

The chromosome paint was obtained as previously described by chromosome flow sorting [21], followed by degenerate oligonucleotide-primed PCR amplification [22]. The flow-sorted probe was labeled with biotin-16-dUTP, and *in situ *hybridization of the probe was performed in a 5 μl volume. The mixture was precipitated and dissolved in 14 μl of hybridization buffer (formamide 50%, dextran sulfate 10%, 2 × SSC). The probe was denatured at 80°C for 10 minutes and re-annealed at 37°C for 90 minutes before hybridization.

#### In situ hybridization

The previously prepared slide was denatured in 70% formamide/2 × SSC, at 65°C for 80 sec, and quenched in an ice-cold 70% ethanol followed by dehydration in 70%, 90%, and 100% ethanol series at room temperature. Hybridization was carried out in a humidified chamber at 37°C overnight. Slides were washed and counterstained with diamidino-2-phenylindole (DAPI, 0.8 ng/μl) for 10 minutes and the slides were mounted with antifade.

#### Microscopy

Analyses were performed under an Axioplan 2 (Zeiss) fluorescence microscope coupled with a CCD camera (Photometrics), and images were captured with FISHview 4.5 software (Applied Spectral Imaging Inc., Vista, CA, USA).

### Quantitative real-time PCR (RT-PCR)

Total RNA was isolated from primary human monocytes/macrophages or humanized glands using the RNeasy kit (Qiagen) according to the manufacturer's instructions. RNA was reverse transcribed using MMLV reverse transcriptase (Invitrogen) and primed with oligo-dT and random hexamers (Invitrogen). The cDNA was amplified using gene-specific primers for CD14, CD68, acyl-malonyl condensing enzyme 1 (AMAC1)-1, IL-10, transforming growth factor (TGF)-β, IL-1β, TNFα, beta-2 microglobulin (β2M), and GAPDH (Additional File 2, Table S2) and 2 × Brilliant II Sybr Green QPCR Mastermix (Stratagene, La Jolla, CA, USA). RT-PCR data were analyzed via the comparative CT (^ΔΔ^CT) method [23]. Four independent cell isolations from different patient samples were used for each experiment.

### Generation of lentiviral particles and transduction of macrophages

GFP-expression lentiviral particles were generated using the PACKH1 kit and 293TN cells (both SBI System Biosciences, Mountain View, CA, USA). Briefly, 293TN cells were plated in a 100 mm tissue culture dish (BD Falcon, Franklin Lakes, NJ, USA) in DMEM (high glucose, Invitrogen) supplemented with 10% FBS such that the cells grew to 70% confluence overnight. The medium was then replaced with DMEM (high glucose) supplemented with 1% FCS; the plasmid mix composed to the manufacturer's instructions was added, and the cells were incubated at 37°C, 5% CO_2 _in a humidified atmosphere overnight. The following day, the cell culture medium containing plasmid was removed; cells were suspended in RPMI supplemented with 10% FCS, and passaged into a 150 mm tissue culture dish (BD Falcon). The virus-containing tissue culture supernatant was collected three days later, filtered (0.45 μm, polyvinylidene fluoride (PVDF); Millipore, Billerica, MA, USA) to remove 293TN cells and was used immediately or stored at -70°C until use. Freshly isolated macrophages were treated with 8 μg/ml polybrene for 10 minutes prior to the addition of virus-containing tissue culture supernatant (1.0 ml supernatant per 3 mls growth medium). Cells were incubated with the supernatant for 36 hr, and then media were replaced with growth medium. Three days post-transduction, green fluorescent protein (GFP) expression was visually confirmed using a fluorescence microscope.

### Proliferation and invasion assays

For all three assays, 24-hr macrophage-conditioned media were collected from macrophages cultured in RPMI containing 2% charcoal-stripped serum +/- estrogen (10^-10 ^M) and used for fibroblast treatment.

Proliferation assays: primary human breast fibroblasts or immortalized human breast fibroblasts proliferating in log phase were placed in RPMI containing 2% charcoal-stripped serum (control media) for 24 hr followed by treatment with either control media +/- estrogen (10^-10 ^M) or macrophage-conditioned media treated +/- estrogen, 10 ng/ml TNFα (Peprotech, Rocky Hill, NJ, USA), 1 or 10 μg/ml Etanercept (Immunex Corporation, Thousand Oaks, CA, USA) alone or in combination with macrophage-conditioned media treated +/- estrogen. Cells were treated for three days, then trypsinized and counted. Data represent four independent experiments with different fibroblasts and macrophage isolations used for each assay.

Invasion assays: BD Biocoat Matrigel invasion chambers (8 μm pores) were used according to the manufacturer's protocol (BD Biosciences, Bedford, MA, USA). Briefly, 125,000 fibroblasts were plated in serum-free media in the top chamber of transwell inserts and were allowed to invade through the membrane for up to 16 hr towards either control media +/- estrogen (10^-10 ^M), or macrophage-conditioned media, or conditioned media from macrophages treated with estrogen in the bottom chamber. Following invasion, the cells were wiped from the top surface of the membrane; the remaining cells were fixed in methanol and stained with a 1% toluidine blue solution. Five independent experiments, each with different patient samples (fibroblasts and macrophage conditioned media) were performed with each individual experiment plated in triplicate to ensure repeatability. Cells were imaged and quantified using NIH Image J 64 software.

For MMP inhibitor invasion studies, 20 or 100 nM MMP9 inhibitor (Calbiochem, Gibbstown, NJ, USA) were added to the top chamber of transwell inserts at the time of plating.

### Quantitative multiplex cytokine and chemokine ELISA assays

A multiplex ELISA kit (Quansys Biosciences, Logan, UT, USA) was used to quantify a panel of 16 cytokines (IL-1α, IL-1β, IL-2, IL-4, IL-5, IL-6, IL-10, IL-12p70, IL-13, IL-15, IL-17, IL-23, IFN-γ, TNF α and TNF β) and 16 chemokines (Eotaxin, GROa, I-309, IL-8, IP-10, monocyte chemoattractant protein (MCP)-1 and -2, regulated on-activation normal T cells expressed and excreted (RANTES), thymus and activation-regulated chemokine (TARC), angiopoietin (ANG)-2, basic fibroblasts growth factors (bFGF), hepatocyte growth factor (HGF), platelet derived growth factor (PDGF)-BB, tissue inhibitor of metalloproteinase (TIMP)-1 and -2, and vascular endothelial growth factor (VEGF) from conditioned media according to the manufacturer's protocol.

### Zymography and western analysis

Conditioned media were collected, concentrated using Amicon Ultra centrifugation filter (MWCO 10K, Millipore), and protein concentrations was determined by Bradford assay (Thermo Scientific; Rockford, IL) according to the manufacturer's instructions.

Zymography: Gelatin zymography was performed using pre-cast 10% Zymogen 0.1% gelatin gels (Invitrogen) according to the manufacturer's instructions. MMP activity was visualized as clear bands against a dark blue background using SimplyBlue SafeStain (Invitrogen) where the protease has digested the substrate. Identification of MMPs was based on the migration pattern of pro- and activated MMP proteins similarly separated, as well as by their published molecular weights.

Western blotting: equal concentrations of conditioned media, as determined by the Bradford assay, were separated by SDS-PAGE under reducing conditions. Membranes were blocked in 5% non-fat milk for 1 hr at room temperature, incubated with primary antibody (MMP9, MMP2; Abcam) overnight at 4°C, washed, and incubated with the appropriate secondary antibody conjugated to horseradish peroxidase (GE Healthcare, Piscataway, NJ, USA). Peroxidase activity was detected using the enhanced chemiluminescence detection system (ECL Plus, GE Healthcare) according to the manufacturer's recommendations. Four independent experiments, each with different patient samples for each cell type with each individual experiment repeated in duplicate, were performed to ensure repeatability for each assay.

### Immunohistochemistry

Immunohistochemistry was performed with appropriate controls as described previously [24]. Briefly, 5 μm thick sections of formalin fixed, paraffin embedded tissue were prepared from humanized glands. The human specific CD14 antibody (1:200, Sigma) was used to detect human monocytes/macrophages in humanized glands, the human-specific proliferating cell nuclear antigen (PCNA) antibody (AbCam ab53048) was used to detect proliferating human fibroblast in the mammary gland, and the human-specific MMP antibody (1:200 Abcam AB76003) was used to detect human cell-derived MMP9. Histosections were pretreated with Dako Target Retrieval solution and stained using Vectastain ABC kit (Vector Laboratories; Burlingame, CA, USA) according to the manufacturer's instructions. Color was developed with diaminobenzidine peroxidase substrate kit (Vector Laboratories) and sections were counterstained with hematoxylin. A minimum of five glands per treatment group was analyzed for each assay.

### Image quantitation

For determination of the percent of gland humanized, H&E-stained sections of each gland were imaged with the Zeiss SteREO Discovery V12 microscope. Total and humanized areas were determined by histological examination, measured using the freehand outline tool, and calculated using Axiovision V4.8 software. Cell invasion was imaged using an Olympus IX51 microscope at 20× magnification and quantified using NIH Image J software (threshold-standardized; measurement determined as percent area: red).

### Statistical analysis

Data were evaluated for significance using *t*-tests or one-way analysis of variance (ANOVA) with the appropriate post hoc analysis (Tukey, Bonferroni) using GraphPad InStat Software version 3.0b (San Diego, CA, USA). Data were considered significant at *P *< 0.05.

## Results

### Murine mammary gland humanization is significantly enhanced with macrophages and ectopic estrogen stimulation

Macrophages perform a vital role in mammary development and function [7]; therefore, we hypothesized that the introduction of human macrophages would enhance humanization of the murine mammary gland. Moreover, the addition of macrophages would permit an intimate look at human immune and stromal-derived paracrine cell interactions in a dynamic *in vivo *model.

The standard humanization procedure does not require ectopic estrogen hormonal stimulation for ductal development of the human epithelial cells as long as the procedure is performed prior to the end of puberty [6]. However, circulating estrogen levels in the mouse are lower compared to circulating levels in premenopausal women, and are essentially comparable to levels found in postmenopausal women [25]. Additionally, macrophages have estrogen receptors [26,27], and estrogens are known to influence the maturation and function of macrophages, as well as to have suppressive effects on the expression of cytokines and other inflammatory modulators [27]. Thus, as the fibroblasts and macrophages used in this study were obtained from premenopausal women, and it is unknown what contributing factor estrogens have on mammary macrophage function in this assay, the effects of ectopic estrogen stimulation in the humanization procedure were also investigated.

Both primary breast fibroblasts and peripheral blood monocytes/macrophages were isolated from premenopausal women. It was taken care that additional, contaminating immune cells were removed via cell culture selection through monocyte and macrophage adherence to plastic. To ensure purity and to obtain a more homogeneous cell population, cells were further differentiated into macrophages by IFNγ and LPS treatment. Additional File 3, Figure S1A shows cells immediately post-isolation and three days post differentiation treatment. Treatment induced a predominately classic activation of macrophage differentiation, as determined by quantitative RT-PCR of genes associated with the classical and alternative activation status of macrophages (Additional File 3, Figure S1B). A comparison of treatment with CSF-1 (100 ng/ml) and IFNγ/LPS was performed as reports suggest that differentiation with CSF-1 in mammary tissue may differentially influence macrophage phenotype. Of the 43 macrophage differentiation and polarization genes analyzed, only four genes were significantly differentially expressed (Additional File 1, Table S1, *P *< 0.05; *BIRC3*, *CCR7*, *CD163*, and *PTX3*), suggesting no substantial difference between differentiation protocols.

**Figure 1:**
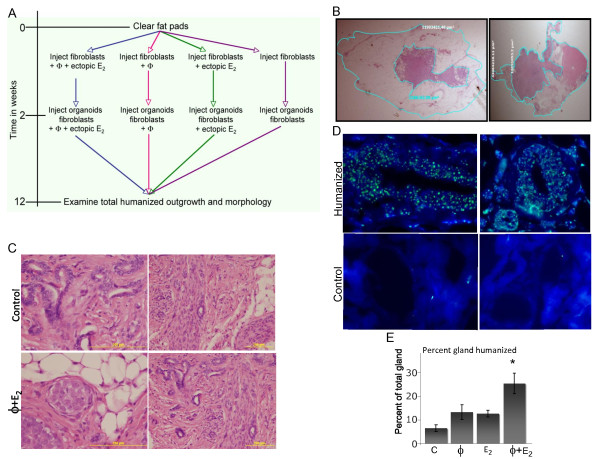
**Human macrophages enhance humanization of the murine mammary gland**. Murine mammary glands were humanized +/- the addition of primary human macrophages and exogenous estrogen supplementation. Humanization was repeated in two independent experiments using different patient/donor-derived normal breast fibroblasts and macrophages between experiments. The primary breast epithelial cells and organoids used for all experiments originated from the same patient. A minimum of ten glands from each treatment group was used for analyses. **(A) **Schema representing steps of procedure. **(B) **Total and humanized areas were determined after two months of growth by histological examination of H&E-stained tissue, imaged with the Zeiss SteREO Discovery V12 and calculated using Axiovision V4.8 software. **(C) **H&E-stained humanized histosections. Images were captured at 40× (left) and 20× (right) magnification. **(D) **Fluorescence *in situ *hybridization (FISH) analysis of humanized mammary glands. Paraffin-embedded histosections of humanized mammary glands were subjected to FISH analysis to confirm the species of origin for the identified regions of humanization. Human X chromosome probe was labeled with biotin-16-dUTP (green). Slides were counterstained with diamidino-2-phenylindole (DAPI, blue) and mounted with antifade. Analysis was performed using the Zeiss Axioplan 2 fluorescence microscope coupled with a CCD camera and images were captured with FISHview 4.5 software at 100× magnification. **(E) **Data represent percent humanization ± standard error (SE) of two independent experiments. A minimum of ten glands per treatment group were measured. *P < 0.05. FB, fibroblast conditioned media; ϕ, macrophage conditioned media; E_2_, estrogen treatment.

In order to study the effect of macrophages and estrogen on mammary gland engraftment and outgrowth in a humanized mouse model of the mammary gland, we cleared the mammary fat pad, injected fibroblasts and macrophages in the presence or absence of ectopic estrogen, and allowed engraftment of the fat pad with these human stromal cells. Two weeks post-humanization, primary breast epithelial cells/organoids and additional fibroblasts were injected into the glands [6] with or without the addition of macrophages corresponding to the initial injections (Figure 1A).

The percent of gland humanized was calculated by histological examination of the identified humanized regions followed by measurement of the total and humanized areas using Axiovision V4.8 software (Figure 1B). In agreement with the standard protocol, the fibroblasts outgrowths and epithelial acini-like structures within the fibroblasts were readily detected in the murine mammary tissue for all treatments tested (Figure 1C). FISH analysis determined that the cellular outgrowths measured/observed were of human origin (Figure 1D). The combined treatment of macrophages and ectopic estrogen stimulation significantly enhanced the percentage of the total gland humanized (Figure 1E, P < 0.05) as well as enhanced the success rate of engraftment and outgrowth (45% vs. 70% glands displaying outgrowths, data not shown). The glands humanized with either macrophages or ectopic estrogen treatment alone exhibited a trend to larger humanized outgrowths compared to the standard humanization protocol. Overall these data suggest that both the percent of the gland humanized as well as the engraftment rate was significantly enhanced by the combined addition of macrophages and ectopic estrogen supplementation.

### Transitory macrophages enhance fibroblast outgrowth during early stages of humanization

Having demonstrated enhanced engraftment and humanization of the mouse mammary fat pad by exposure to estrogen and human macrophage injection, without observing an altered histology of the humanized areas between different treatment groups, we next asked if a combined macrophage/estrogen treatment might influence overall cellular growth or enhance the invasiveness of human fibroblasts.

**Figure 2:**
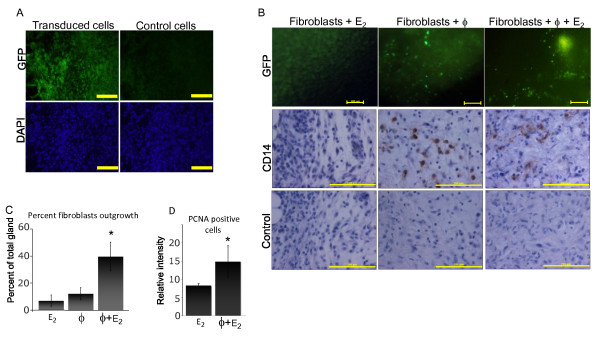
**Transitory macrophages enhance early states of fibroblast outgrowth during humanization**. **(A) **Primary human monocytes/macrophages were isolated via Ficoll separation from apheresis product, differentiated, and then transduced with green fluorescent protein (GFP). Images show cells 48 hr post-transduction with GFP or negative controls. Cell nuclei were stained with diamidino-2-phenylindole (DAPI) for visualization (blue, bottom panels). **(B) **Primary breast fibroblasts were injected with or without the GFP-expressing macrophages in the presence or absence of ectopic estrogen. Six days post-injection freshly excised glands were imaged using a GFP filter. Immunohistochemical detection of human macrophages (day six) humanized glands using a human-specific CD14 antibody (top panels) or corresponding negative controls (bottom panels; scale bar = 200 μm). **(C) **Percent gland humanized after ten days of fibroblast growth +/- estrogen +/- macrophages. Total and humanized areas were determined by histological examination, imaged with the Zeiss SteREO Discovery V12 and calculated using Axiovision V4.8 software. Data represent percent humanization ± SD. Four glands per treatment group were measured. *P < 0.01. **(D) **Tissues were subjected to immunohistochemical analysis with a proliferating cell nuclear antigen (PCNA) antibody to identify proliferative cells. Stained histosections were imaged and quantified using NIH Image J software. Data represent average of four counts per gland ± SD. Three glands per treatment group were measured. *P < 0.02.

Immunohistochemistry using a human-specific CD14 antibody revealed that no human macrophages were present at the time of gland collection (two months post-final injection of cells, data not shown). Therefore, we examined the timing of the disappearance of the human macrophages from the gland using freshly isolated macrophages transduced with lentiviral particles to induce expression of GFP (Figure 2A). Glands were humanized and collected 2, 6, 10 and 40 days post-injection. Presence of macrophages was measured by three methods: immunofluorescent detection of GFP immediately following excision of the glands, immunohistochemical detection using a human-specific CD14 antibody on paraffin-embedded histosections, and RT-PCR of RNA isolated from the glands using human specific primers to CD14 and CD68.

Macrophages were readily detected two days post-injection by all three methods. RT-PCR analysis showed a decrease of human CD14- and CD68-mRNA between day 2 and day 6 after injection of human macrophages into the mammary fat pad (Table 1). Human CD14 and human CD68 were no longer detectable 10 days after injection of macrophages; GFP-labeled human macrophages were also readily detected in the mammary fat pad 2 days and 6 days after inoculation with GFP-labeled macrophages (Figure 2A, B). Likewise, we demonstrated the presence of human macrophages in the mammary fat pad 6 days after inoculation by immunohistochemistry for human CD14 (Figure 2B). At days 24 and 40, all methods confirmed the lack of human macrophages within the humanized gland (data not shown). Taken together, these data demonstrate that human macrophages are only temporarily present in the inoculated mammary fat pad. This indicates a transient interaction of macrophages, fibroblasts, and the stroma of the mammary fat pad during the first stage of mammary fat pad humanization, which subsequently enhances future engraftment with human breast epithelial cells.

**Table 1:** Time course of real time-PCR detection of human macrophages in humanized glands

	CD14	SD	CD68	SD	GAPDH	SD
**Day 2**						
+ E_2_	No CT		No CT		22.28	0.26
+ Macrophages	23.88	0.10	28.18	0.24	21.59	0.24
+ E_2 _and Macrophages	26.36	0.16	28.52	1.50	22.69	0.23
**Day 6**						
+ E_2_	No CT		No CT		22.48	0.31
+ Macrophages	28.37	3.40	30.02 ^a^	1.39	20.88	0.70
+ E_2 _and Macrophages	27.65	4.92	30.01^a^	3.57	19.69	0.91
**Day 10**						
+ E_2_	No CT		No CT		19.16	0.33
+ Macrophages	31.05^a^	0.81	30.81^a^	1.55	18.51	0.15
+ E_2 _and Macrophages	29.43	1.51	31.28 ^a^	1.12	18.65	1.13
**Controls**						
Human monocytes	14.4	0.14	16.3	0.16	20.59	0.07
Human macrophages	16.5	0.16	20.2	0.09	21.00	0.20
Murine mammary gland	No CT		No CT		21.45	0.14

^a^Cycle Threshold (CT) values over 30 were not considered positive for a detectable signal. Analysis was terminated at 32 cycles. E_2_, ectopic estradiol treatment; GAPDH, glyceraldehyde-3-phosphate dehydrogenase.

To test this hypothesis, glands were humanized with fibroblasts and ectopic estrogen, with fibroblasts and macrophages, or with a combination of all three to evaluate the initial step of humanization, the outgrowth of fibroblasts into the murine mammary fat pad. After ten days of growth, the glands were harvested as in the standard protocol for the initial humanization step. As shown in Figure 2C, the combination of treatments significantly enhanced fibroblast growth throughout the gland compared to the addition of macrophages or ectopic estrogen alone (*P *< 0.01). These data suggest that the enhanced humanization is due to an increase in the overall area of human stromal outgrowth, thereby providing a larger humanized area for acini proliferation and outgrowth. Indeed, immunohistochemistry revealed significant increase of PCNA-positive, proliferating fibroblasts in glands humanized with macrophages compared to fibroblasts alone (Figure 2D, P < 0.02).

Collectively, these data indicate that the stimulatory mechanisms of the macrophages on humanization are transitory, as the macrophages disappear from the murine gland within 10 days post-injection. However, our data indicate that their presence at the time of injection, and within a week thereafter, has a significant and long lasting effect on mouse mammary gland humanization with human fibroblasts and consecutive engraftment with human mammary epithelial cells. The increased proliferation of human fibroblasts might be a relevant mechanism for the observed increased humanization of the mouse mammary fat pad.

### Macrophages enhance fibroblast proliferation, in part, through a TNFα dependent mechanism

**Figure 3:**
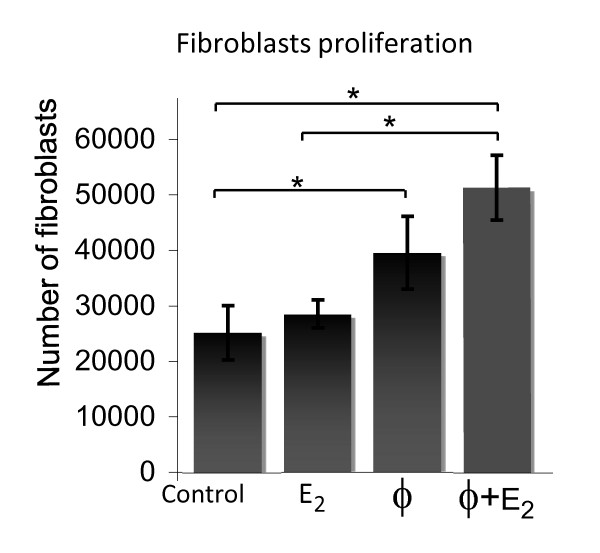
**Macrophages stimulate primary breast fibroblast proliferation**. Primary human monocytes/macrophages were isolated, differentiated, treated +/- estrogen (10^-10 ^M) for 24 hr and conditioned media were collected. Primary human breast fibroblasts were placed in 2% charcoal-stripped serum-containing media 24 hr prior to treatment +/- estrogen or with macrophage-conditioned media +/- estrogen for three days, then trypsinized and counted. Data represent four independent experiments with different fibroblasts and macrophage isolations used for each assay. *P < 0.01. ϕ, macrophage conditioned media; E2, estrogen treatment.

We determined that paracrine interactions between primary macrophages and fibroblasts enhance humanization of the gland. To identify potential mechanisms by which the macrophages enhanced humanization, four different *in vitro *cell proliferation assays were used to determine if (1) direct cell-to-cell contact was necessary between the fibroblasts and macrophages, or (2) if secreted factors from the macrophages were sufficient to induce fibroblasts proliferation, and (3) whether estrogen enhanced these effects. Fibroblasts were either co-cultured directly with macrophages in the presence or absence of estrogen treatment, cultured in the presence of the macrophages but separated from physical contact with a transwell insert, treated with macrophage-conditioned media, or treated with co-conditioned media from fibroblast and macrophage direct co-cultures (Additional File 4, Figure S2). Overall, when fibroblasts were cultured in the presence of the estrogen-treated macrophage-conditioned media, cell proliferation was equivalent or greater than any other treatment condition. Based on these initial results, we more rigorously examined the effects of macrophage-conditioned media in the presence or absence of estrogen treatment using seven freshly isolated individual fibroblasts patient samples each treated with the conditioned media from three different macrophage patient samples (Figure 3). These data confirm the original observation that fibroblasts cultured in the presence of the estrogen-treated macrophage-conditioned media exhibit significantly enhanced cell proliferation compared to control or estrogen treatment (*P *< 0.01).

**Figure 4:**
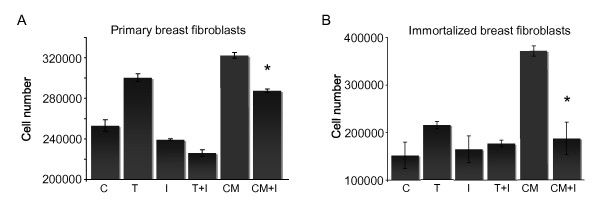
**Macrophage-secreted TNFα enhances breast fibroblast proliferation**. Primary human monocytes/macrophages were isolated, differentiated, treated with estrogen (10^-10 ^M) for 24 hr and conditioned media (CM) were collected. Primary human breast fibroblasts were placed in 2% charcoal-stripped serum-containing media 24 hr prior to treatment with 2% charcoal-stripped serum-containing media + estrogen (10^-10 ^M), media + 10 ng/ml TNFα (T), + TNFα inhibitor (I), the combination TNFα + inhibitor (T+I), macrophage-conditioned media (CM) or CM + TNFα inhibitor (CM+I). For each assay, cells were treated for three days then trypsinized and counted. Data shown are from one representative experiment of two independent experiments. *P < 0.05 from CM.

To identify macrophage-specific secreted factors responsible for the increase in fibroblast proliferation, quantitative multiplex cytokine and chemokine ELISA-based assays were performed. Three independent monocyte/macrophage isolations were prepared, followed by differentiation and treatment for 24 hr in the presence or absence of estrogen. Half of the macrophage-conditioned media were immediately frozen and stored for analysis, and half were placed on fibroblast cultures for 24 hr to obtain co-conditioned media. Each of the three macrophage conditioned media samples were used to treat a minimum of two independent breast fibroblast isolations and the co-conditioned media were collected from each individual fibroblast sample. One striking observation was a significant decrease in TNFα after macrophage-conditioned media was used to treat the fibroblasts (mean ± SD, 60.9 ± 1.1 pg/ml vs. 17.6 ± 2.8 pg/ml, respectively; *P *< 0.001), potentially suggesting cellular uptake. TNFα has been reported to stimulate dermal and foreskin fibroblast chemotaxis and proliferation *in vitro *and *in vivo *[28-30]; therefore, we tested the ability of the macrophage-derived TNFα to stimulate breast fibroblast proliferation. Initial studies were performed using hTERT immortalized breast fibroblasts [6] to determine whether breast fibroblasts proliferate in response to TNFα stimulation, and to optimize the concentration of TNFα inhibitor (Etanercept) (data not shown). Immortalized and primary breast fibroblasts were then treated with estrogen-stimulated macrophage-conditioned media or TNFα (10 ng/ml) in the presence or absence of the TNFα inhibitor (10 μg/ml). As shown in Figure 4A and 4B, the proliferation of breast fibroblasts treated with estrogen-stimulated macrophage-conditioned media was significantly inhibited in the presence of the TNFα inhibitor (*P *< 0.05). Macrophage-conditioned media enhanced fibroblast proliferation more than TNFα alone (Figure 4B), suggesting that other factors also contributed to cell proliferation; however, our data demonstrate that enhanced proliferation occurs, in part, through a TNFα dependent mechanism.

### Macrophages stimulate enhanced fibroblast invasion through upregulation of MMP-9 activity

**Figure 5:**
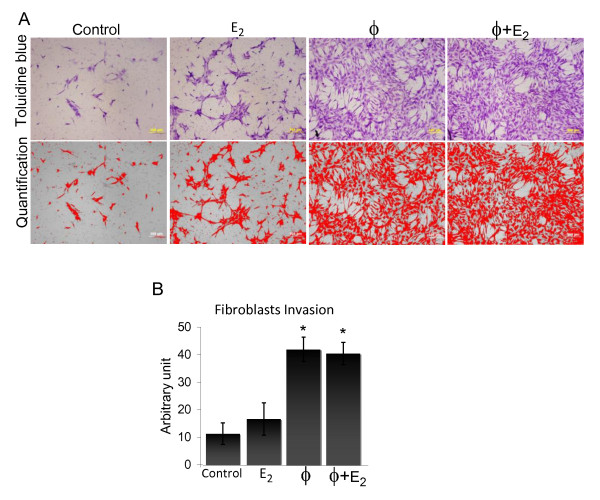
**Enhanced primary breast fibroblast invasion in the presence of human macrophages conditioned media**. Primary human macrophages were treated +/- estrogen (10^-10 ^M) in 2% charcoal-stripped serum-containing media and 24 hr-conditioned media were collected. Primary human breast fibroblasts were seeded in serum-free media into Matrigel invasions chambers in triplicate. Chemoattractants were 2% charcoal-stripped serum (control), control media + estrogen (10^-10 ^M), macrophage-conditioned media, or conditioned media from macrophages treated with estrogen. **(A) **24 hr post-seeding, invaded cells were stained with toluidine blue (top panels), imaged and quantified using NIH Image J software (measurement highlighted in red, bottom panels). **(B) **Data represent average area measurement of invaded cells +/- standard error (SE) of five independent experiments. *P < 0.01 above control or E_2 _treatment. ϕ, macrophage conditioned media; E_2_, estrogen treatment.

During humanization, the fibroblasts injected into the gland must proliferate as well as migrate throughout the gland, which requires the active degradation of the basement membrane and extracellular matrix of the murine gland. Having already determined that macrophages enhanced fibroblast proliferation, we next investigated whether stimulation by macrophages, estrogen, or the combination, enhanced fibroblast invasion as well. Primary human breast fibroblasts were subjected to transwell Matrigel invasion assays using either control or macrophage-conditioned media containing 2% charcoal-stripped serum +/- estrogen (10^-10 ^M) as chemoattractants. Twenty-four hours post-seeding, invaded cells were stained with toluidine blue, imaged, and quantified (Figure 5A). Results from five independent experiments using different fibroblast and macrophage isolations for each assay demonstrated that both macrophage-conditioned media and estrogen-stimulated macrophage-conditioned media significantly increase fibroblast invasion above controls (Figure 5B, P < 0.01). Estrogen alone enhanced fibroblast invasion, but the increase was not significant. These data demonstrate that chemokines and additional molecules secreted from the macrophages enhance extracellular matrix degradation and fibroblasts invasion, potentially replicating another mechanism by which macrophages enhance humanization *in vivo*. These results were similar to those obtained in the *in vitro *proliferation assays; either macrophages alone or estrogen-stimulated macrophages were able to significantly enhance fibroblast behavior. In contrast to these *in vitro *measurements of cell behavior, ectopic estrogen stimulation is required to obtain the most significant amount of humanization *in vivo*.

**Figure 6:**
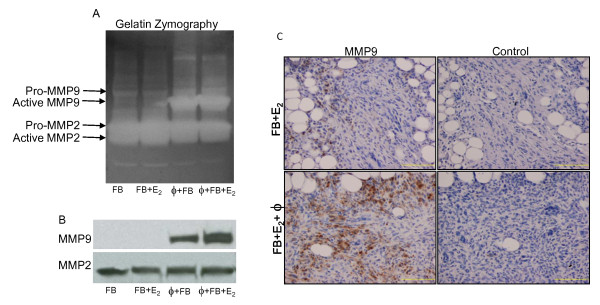
**Primary macrophage conditioned media stimulates primary breast fibroblast matrix metalloproteinase (MMP)-9 secretion**. Primary human macrophages were treated +/- estrogen (10^-10 ^M) in 2% charcoal-stripped serum-containing media and 24 hr-conditioned media were collected. Primary human breast fibroblasts were placed in 2% charcoal-stripped serum-containing media 24 hr prior to treatment with 2% charcoal-stripped serum, control media + estrogen (10^-10 ^M), macrophage-conditioned media, or conditioned media from macrophages treated with estrogen. **(A) **Gelatin zymography. **(B) **Western blot analysis. Results are representative of a minimum of three independent experiments with different fibroblasts and macrophage isolations. **(C) **Murine mammary glands were humanized and +/- the addition of primary human macrophages and exogenous estrogen supplementation. Immunohistochemical analysis using a human-specific MMP9 antibody of paraffin-embedded histosections glands six days post-humanization. Scale bar = 200 μM. FB, fibroblast conditioned media; ϕ, macrophage conditioned media; E_2_, estrogen treatment.

Given the known role of matrix metalloproteinases (MMPs) in cell invasion and mammary development [31,32], we hypothesized that upregulation of MMPs was one mechanism by which macrophages enhanced fibroblasts invasion and thus overall humanization. Gelatin zymography and western blot analyses were used to evaluate the levels and activity of MMPs from the conditioned media of either fibroblasts alone or stimulated with macrophage-conditioned media. Membranes were stained with Ponceau S to ensure equal loading of protein (Additional File 5, Figure S3). Four independent experiments, each with different patient samples for each cell type with each individual experiment repeated in duplicate, were performed to ensure reproducibility for each assay. Results demonstrate that fibroblasts treated with macrophage-conditioned media had significantly increased MMP9 activity and expression (Figure 6A, B) while MMP2 levels remained relatively unchanged regardless of treatment. An additional increase in MMP9 activity and levels in the conditioned media of fibroblasts treated with the estrogen stimulated macrophage-conditioned media was also observed (Figure 6A, B; quantification in Additional File 5, Figure S3).

To confirm our *in vitro *findings, the levels of MMP9 during early stages of humanization were examined. Paraffin-embedded sections of glands humanized with fibroblasts and ectopic estrogen or fibroblasts with primary human macrophages and ectopic estrogen were collected 2, 6 (Figure 6C), and 10 days post-injection and analyzed immunohistochemically using a human-specific MMP9 antibody. Analysis at each time point expressed significantly higher levels of MMP9 in the humanized regions of the gland, similar to results from zymography and western analyses, demonstrating an increase in MMP9 when macrophages are present.

**Figure 7:**
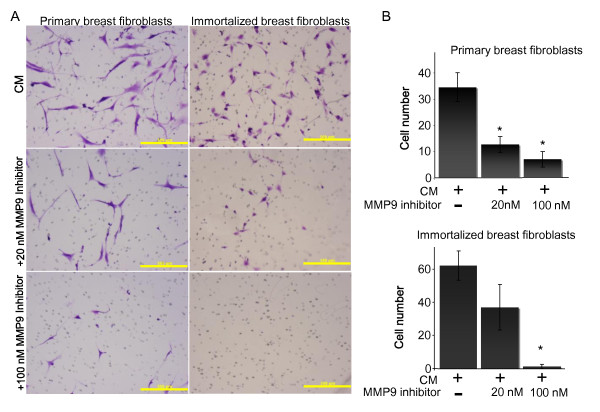
**Macrophage-conditioned media-enhanced breast fibroblast invasion requires matrix metalloproteinase (MMP)-9**. Primary human macrophages were treated +/- estrogen (10^-10 ^M) in 2% charcoal-stripped serum-containing media and 24 hr-conditioned media were collected. Primary human breast fibroblasts were seeded in serum-free media into Matrigel invasion chambers in triplicate. Chemoattractants were conditioned media (CM) from macrophages treated with estrogen (10^-10 ^M) +/- the indicated amount of MMP9 inhibitor. **(A) **24 hr post-seeding, invaded cells were stained with toluidine blue; scale bar = 500 μm. **(B) **Data represent average number of invaded cells +/- SD; four areas were measured per chamber. *P < 0.01.

To further support our hypothesis that increased activity of MMPs enhances humanization, we performed additional assays investigating the ability of macrophage-stimulated MMP9 activity to increase fibroblast invasion. Primary and immortalized human breast fibroblasts were again subjected to transwell Matrigel invasion assays using estrogen-stimulated macrophage-conditioned media as the chemoattractant; however, a specific MMP9 inhibitor was added to the fibroblasts when plated in the transwell chamber. Invaded cells were stained with toluidine blue 24 hr post-seeding, imaged, and quantified (Figure 7A). Results from two independent experiments using different fibroblasts and macrophage isolations for each assay demonstrated that inhibition of MMP9 activity decreased fibroblast invasion in a dose-dependent manner (Figure 7B, P < 0.01). However, it is noted that in these *in vitro *studies it is not possible to distinguish chemotaxis from chemokinesis. Collectively, our results demonstrate that macrophage-stimulated enhanced humanization is due in part, to increased MMP9 degradation of extracellular matrix, thereby facilitating fibroblast invasion throughout the gland.

### Macrophages stimulate enhanced tumor formation in humanized glands

**Figure 8:**
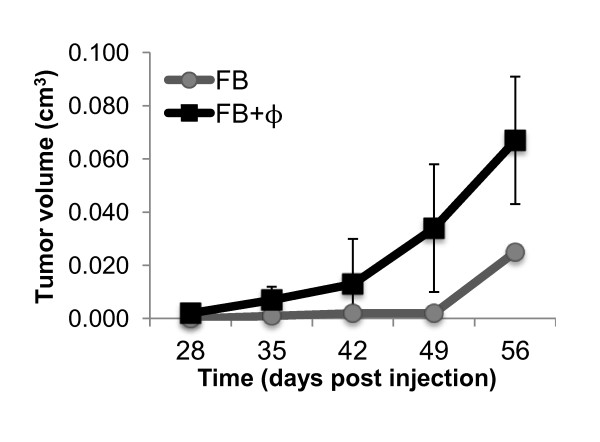
**Macrophages enhanced tumor growth of humanized glands**. Murine mammary glands were humanized +/- the addition of primary human macrophages and exogenous estrogen supplementation (5 mice per treatment group). Two weeks post-humanization, 1 × 10^6 ^T47D breast cancer cells were injected with fibroblasts or fibroblasts and macrophages into the humanized region. One tumor out of a total of five injected mice developed tumors in the standard humanized treatment group, while three tumors out of five injected mice developed tumors in the glands humanized with macrophages. Data represent mean +/- SD.

Previous studies show that the source of stromal fibroblast can significantly affect epithelial cell morphology and proliferation, as well as tumor engraftment and aspects of behavior [6,33-39]. Therefore, an initial study was performed to observe the effect of macrophages on breast cancer cell engraftment of humanized glands. This experiment is one initial experiment, used only to support the need for future tumorigenesis studies using this model. The right abdominal gland was humanized in the presence or absence of estrogen-stimulated macrophages, and mice were supplemented with subcutaneous estrogen pellets (five mice were injected in the control group and five mice in the treatment group). Two weeks post-humanization, 1 × 10^6 ^T47D breast cancer cells were injected with fibroblasts or fibroblasts and macrophages into the humanized region. As shown in Figure 8, the glands humanized with macrophages had enhanced engraftment rate and tumor growth compared to the glands humanized with fibroblasts alone (one tumor in the standard humanized glands out of five injected mice vs. three tumors out of five mice in the macrophage group). Initial studies show no overt differences in tumor morphology between treatment groups and additional studies are under investigation. Similar to humanized studies, no human macrophages were detected in the xenograft tumors or surrounding tissues at the time of collection.

## Discussion

This study demonstrates that intricate, temporary, stroma-derived paracrine cell interactions in a humanized *in vivo *model system can improve engraftment of the mammary fat pad. Based on the heterogeneic mouse model of the human mammary gland developed by Kuperwasser *et al. *[6] we expanded upon the model by incorporating human immune cells, in addition to human fibroblasts, to investigate multi-cellular primary human cell interactions *in vivo*. The addition of macrophages enhanced humanization of the gland by augmenting fibroblast proliferation and invasion throughout the gland, thereby providing a larger stromal bed for human breast epithelial cell proliferation and formation of acini and ductal structures. We have identified two specific paracrine mechanisms involved in enhancement of humanization: increased fibroblast proliferation stimulated by macrophage-derived TNFα as well as a macrophage-stimulated increase in MMP9 expression and proteinase activity. Our observed *in vivo *results have been confirmed with *in vitro *studies, again highlighting the value of this model to interchangeably support *in vitro *and *in vivo *results.

It is of note that no gross morphologic differences were observed within the epithelial structures of the macrophage- and estrogen-stimulated humanized outgrowths compared to the standard protocol (Figure 1C). However, one should note that in the present study we did not investigate epithelial function or differentiation during pregnancy and lactation. The critical role macrophages play in orchestrating these important developmental stages of the mammary gland is continually emerging [9,40-42]; future experiments using this *in vivo *model to further observe macrophage function during pregnancy and lactation will be an interesting avenue to explore. The activation stage of macrophages has recently been reported to have a unique function during different stages of mammary gland differentiation [43] and has been shown to differentially influence breast cancer cell behavior [44]. This model system enables novel, *in vivo *studies investigating the role of human macrophages in normal breast, as well as in breast cancer xenograft studies. However, it should be noted that the addition of macrophages may not have an effect on epithelial cell differentiation, as the introduction of M1-type macrophages may have created more of an initial wound healing microenvironment, resulting in improved human fibroblast engraftment during the resolution phase when human macrophages are no longer present.

In the present study, peripheral blood monocytes isolated from premenopausal women were differentiated using the classical method of adherence to plastic followed by treatment with LPS and IFNγ [45,46]. IFNγ is an immunostimulatory cytokine that primes monocytes for differentiation in response to LPS [47]. This method was chosen as it produced a more homogeneous population of macrophages as compared to other differentiation methods, and the macrophages produced higher amounts of proinflammatory cytokines compared to alternatively activated macrophages (Additional File 1, Figure S1). Although limited data exist on the role of proinflammatory cytokines in the human breast, IFNγ has been reported to be present in the breast during development and milk production [48]. *In vitro *studies have shown that human mammary epithelial cells contain the IFNγ receptor and are sensitive to IFN-γ stimulation, resulting in inhibition of proliferation, disruption of cell polarity and tight junctions; all are critical steps in milk stasis, involution and tissue remodeling during outgrowth [49-51]. Therefore, the presence of IFNγ during stages of breast tissue remodeling suggests the potential for monocyte/macrophage exposure in breast tissue, and a role for IFN-γ in breast function. However, many of the studies defining the role of mammary macrophages have focused on the role of CSF-1, which regulates macrophage behavior, morphology and motility and, through a series of eloquent genetic mouse-model experiments, has been shown to be critical for mammary gland development and function [52,53]. Gyorki *et al. *(2009) demonstrated that mammary macrophages contribute to normal stem/progenitor cell function in the developing mouse mammary gland, using both the *Csf1*^op/op ^mice and the clondronate-containing liposomes ablation method [54]. They suggest that mammary stem cells require macrophage-derived factors to be fully functional. Our data show that *in vitro *treatment of the cells with either CSF-1 or LPS/IFNγ has no significant effect on 39 of the 43 differentiation and polarization genes analyzed (Additional File 1, Table S1), suggesting no substantial difference between differentiation protocols. However, the possibility exists that upon introduction to the mouse mammary fat pad, the local and systemic factors had an additional influence on the human macrophages phenotype. Given the wealth of data on CSF-1 in the developing gland, it would be of interest to observe the effect of CSF-1-differentiated human macrophages on humanization of the mammary fat pad.

In the present study, it is not known whether the macrophages stimulated a wound-healing response or other developmental processes to enhance humanization of the mouse fat pad. The LPS/IFN-γ-stimulated macrophages exhibited increased proinflammatory cytokines compared to freshly isolated macrophages. As shown in an eloquent study by Vaillant *et al. *[55], an increase in growth factors and cytokines can bias the numbers of stem cells present in normal or tumoral tissue, as well as alter progenitor/stem cell proliferation. Thus, it is possible that the secretion of proinflammatory cytokines by the LPS/IFN-γ-stimulated macrophages may have influenced humanization of the glands by stimulating progenitor cell response. Conversely, the increase in cytokine production by the macrophages potentially mimicked a wound response, thereby enhancing the outgrowth of the fibroblasts into the mammary fat pad in this model. Acute wounding was shown to increased tumor growth in a syngeneic mouse breast cancer model and, moreover, the effect of accelerated tumor growth due to wounding could be mimicked by acellular wound fluid [56]. We evaluated whether the effects of the macrophages on humanization could be stimulated using acellular-conditioned media (Additional File 6, Figure S4); however, concentrated macrophage-conditioned media were not capable of enhancing fibroblast humanization throughout the mammary gland. This suggests that either the physical presence of the macrophage in the mammary gland was necessary for enhanced humanization, or that human macrophage interactions with the murine stromal cells were necessary for the enhanced humanization observed.

Macrophage cytokine and chemokine production is reportedly suppressed by estrogen, often mediated by transcriptional or nongenomic repression of gene expression [27]. In the present study, estrogen had no effect on TNFα secretion (60.9 ± 1.1 vs. 60.1 ± 2.1 pg/ml); however, estrogen treatment alone or in combination with macrophage-conditioned media significantly decreased the concentration of the chemokines IL-8 and IL-23, as well as the chemokine Eotaxin (Additional File 7, Table S3, *P < 0.05). We hypothesize that the estrogen-stimulated additive effects on enhanced fibroblast humanization of the gland are due to combinatory effects of estrogen-directed suppression of macrophage-derived inhibitory cytokines/chemokines, as well as estrogen-stimulated proliferation in combination with other growth factors *in vivo*. The extensive and complex mechanisms of estrogen signaling are known to have a multitude of effects on breast cell growth and function [57-60]. Therefore, it is not surprising that exogenous estrogen augments the macrophage-stimulated proliferation of steroid-sensitive breast stromal cells in this *in vivo *model.

In this report we primarily investigated normal human breast cell interactions; however, this model is easily adaptable for studying primary human immune and stromal cell interactions in the breast tumor microenvironment. Many of the signaling mechanisms that control the outgrowth of cells throughout the breast during pubertal development and the continual repopulation of cells during steroid-driven cycles of growth are often exploited by tumor cells to support their parasitic growth and invasive properties. It is well acknowledged that immune cells play a significant and complex role in promoting tumorigenesis and metastasis [17]. Macrophages, in particular, have been shown to migrate to distinct tumor sites and, in response to the varying localized stimuli, release specific growth factors and molecules to regulate cancer cell proliferation, invasion, angiogenesis, and metastasis [13]. Our preliminary study observing the effect of macrophages on tumor formation demonstrated an increase in tumor cell engraftment and growth. Previous studies have shown a suppressive effect of normal fibroblasts on tumor formation [6,33-39], potentially explaining the low efficiency of tumorigenesis in the glands humanized with primary breast fibroblasts. Additionally, as stated above, the high level of cytokine/chemokine production secretion from the macrophages potentially stimulated a wound response or augmented tumor progenitor cell proliferation similar to results shown in other studies [54-56]. It is well documented that inflammation and a wound-like signature promotes breast cancer tumorigenesis and is highly prognostic of breast cancer survival [61-63]. TNFα is a known mediator of the inflammatory response [63], and is a potential candidate for a mediator of the observed increase in tumorigenesis. It was recently shown that TNFα secretion from cultured macrophages was significantly increased after incubation with breast cancer tumor supernatants, and resulted in enhanced tumor cell invasion and adherence to endothelium [64]. Additionally, inhibition of TNFα has been shown to decrease breast cancer cell aggressive behavior, including metastasizing to bone [65]. Moreover, macrophages have been shown to play a key role in breast cancer; tumor-associated macrophages produce factors that promote tumor cell proliferation, migration, angiogenesis, tissue remodeling, reduce the local immune response to tumor cells, and are associated with poor prognosis in breast cancer patients [17,64,66-68]. While further studies are required to fully understand the role of macrophages in the humanized gland, this report presents the prospect of studying primary human macrophage/tumor interactions in a dynamic *in vivo *model; potentially further advancing our knowledge of the tumor-derived signals that promote distinct macrophage behavior.

## Conclusions

Our findings demonstrate intricate immune and stromal cell paracrine interactions in a humanized *in vivo* model system. We confirmed our *in vivo* results with *in vitro* analyses, highlighting the value of this model to interchangeably substantiate *in vitro* and *in vivo* results. It is critical to understand the signaling networks that drive paracrine cell interactions to fully understand the basic mechanisms of breast cell function. Moreover, many of the signaling mechanisms that control the outgrowth of cells throughout the breast during pubertal development and the continual repopulation of cells during steroid-driven cycles of growth are often exploited by tumor cells to support their parasitic growth and invasive properties. This report presents a dynamic *in vivo* model to study primary human immune/fibroblast/epithelial interactions. This model can readily be applied to advance our knowledge of the stromal-derived signals that promote tumorigenesis.

## Abbreviations

ANOVA: one-way analysis of variance; ANG: angiopoietin; bFGF: basic fibroblasts growth factors; β2M: beta-2 microglobulin; EGF: epidermal growth factor; FBS: fetal bovine serum; FCS: fetal calf serum; DAPI: diamidino-2-phenylindole; DMEM: Dulbecco's modified eagle medium; ELISA: enzyme-linked immunosorbent assay; FISH: fluorescence *in situ *hybridization; GAPDH: glyceraldehyde-3-phosphate dehydrogenase; GFP: green fluorescent protein; H&E: haematoxylin and eosin; HGF: hepatocyte growth factor; hTERT: human telomerase reverse transcriptase; IL: interleukin; LPS: lipopolysaccharide; IFN: interferon; MCP: monocyte chemoattractant protein; MMP: matrix metalloproteinase; PCNA: proliferating cell nuclear antigen; PCR: polymerase chain reaction; PDGF: platelet derived growth factor; PVDF: polyvinylidene fluoride; RANTES: regulated on-activation normal T cells expressed and excreted; SDS-PAGE: sodium dodecyl sulfate polyacrylamide gel electrophoresis; TAM: Tumor associated macrophage; TARC: thymus and activation-regulated chemokine; TIMP: tissue inhibitor of metalloproteinase; TNF: tumor necrosis factor; VEGF: vascular endothelial growth factor.

## Competing interests

The authors declare that they have no competing interests.

## Authors' contributions

JMF conceived and designed the study, performed or participated in all experiments, their analyses and interpretation, and wrote the manuscript. TCM and MK participated in macrophage isolation and *in vivo *experiments. EG assisted in data analysis, drafting and editing the manuscript. CHS provided the GFP viral particles and participated in editing the manuscript. DS and MAT performed the comparative PCR expression and analysis of differentiated macrophages. BKV conceived the study, assisted in data analysis and edited the manuscript. All authors read and approved the final manuscript.

## Supplementary Material

Additional file 1 **Supplementary Table 1**. Quantitative real time (qRT)-PCR analysis of IFN/lipopolysaccharide (LPS) vs. colony-stimulating factor (CSF)-1 treatment on genes associated with macrophage differentiation. Click here for file

Additional file 2 **Supplementary Table 2**. Real time (RT)-PCR primer sequences. Click here for file

Additional file 3 **Supplementary Figure 1**. Representative images of macrophages treated in culture and real time (RT)- PCR analysis of the activation stages of macrophages *in vitro*. Click here for file

Additional file 4 **Supplementary Figure 2**. Graphs of macrophage stimulation of primary breast fibroblast proliferation *in vitro*. Click here for file

Additional file 5 **Supplementary Figure 3**. Ponceau staining of membranes, quantitation of zymography and western analysis, and representative images of ImageJ quantitation of stained tissue histosections. Click here for file

Additional file 6 **Supplementary Figure 4**. Graph comparing glands humanized with conditioned media vs. macrophages. Click here for file

Additional file 7 **Supplementary Table 3**. Table of values obtained from conditioned media ELISAs. Click here for file
