# Supplementary material for: Paracrine interactions between primary human macrophages and human fibroblasts enhance murine mammary gland humanization in vivo
Source: Breast Cancer Res. 2012 Jun 25;14(3):R97. doi: 10.1186/bcr3215 (PMC3446360; doi:10.1186/bcr3215)
Supplement: Additional file 1 — Supplementary Table 1. Quantitative real time (qRT)-PCR analysis of IFN/lipopolysaccharide (LPS) vs. colony-stimulating factor (CSF)-1 treatment on genes associated with macrophage differentiation. [file bcr3215-S1.PDF]

Table S1. Comparison of IFNγ/LPS vs. CSF-1 treatment on genes associated with macrophage differentiation.

Gene Symbol	AvgΔCt CSF-1	AvgΔCt LPS+IFN	Comparative P-values
CD11b	-0.8925	-0.2355	0.2193
CD11c	-0.1136	0.3179	0.5704
CD36	-1.9537	1.3400	0.3448
CD68	-2.9596	-3.3430	0.7181
CD163	-3.4273	1.4897	0.0166
CD206	-3.1558	-0.8779	0.1202
CCL19	3.0084	0.6073	0.3331
CCL20	3.5079	1.7113	0.3354
CCR2	5.1343	3.0163	0.4016
CCR7	2.6012	-2.3846	0.0024
CXCL1	-3.4176	-2.9811	0.5352
CXCL3	-1.9285	-1.2965	0.4113
CX3CR1	12.3680	12.1284	0.3949
IL-1b	-2.5948	-2.1312	0.5474
IL-6	-1.6040	-2.2661	0.9862
IL-7R	1.3226	0.2934	0.3340
IL-8	-6.3463	-7.7629	0.9448
IL-12	9.2189	6.1653	0.0518
IL-23	3.0345	1.0819	0.3675
ARG	7.7676	6.0158	0.0667
BCL2A1	-1.2623	-3.0647	0.1035
BIRC3	0.9049	-1.0424	0.0292
COX2	1.3572	-0.2271	0.4762
CSF-1	0.5722	0.2052	0.8845
CSF-2	3.1538	1.6392	0.1042
EDN1	2.7897	3.6288	0.5548
F4/80	2.7759	2.2287	0.5932
FGL2	-2.1475	-1.8672	0.7319
FN1	3.0310	2.5807	0.3753
HS3ST1	3.8242	7.2829	0.0744
HSD11B1	2.4674	1.5985	0.3199
INDO	-2.6050	-6.3067	0.0678
iNOS	10.1832	9.6089	0.4207
LIPA	-4.6238	-2.7631	0.0704
MAF	1.8025	1.4837	0.7381
MMP14	-3.1776	-3.7413	0.8243
MS4A4A	3.2646	2.1769	0.0968
MSR1	-0.3948	2.1323	0.3897
OASL	3.4461	2.3218	0.9124
OPN	-0.9192	1.1796	0.4318
PTX3	5.5128	9.6655	0.0497

TNF-α	1.8735	1.2187	0.2438
VEGF	0.1270	-2.2535	0.2291

AvgΔCt, average delta CT; The p-values are the averaged relative gene expression between GAPDH-normalized CSF-1 versus LPS and INFγ treated cells.
