# Supplementary material for: Paracrine interactions between primary human macrophages and human fibroblasts enhance murine mammary gland humanization in vivo
Source: Breast Cancer Res. 2012 Jun 25;14(3):R97. doi: 10.1186/bcr3215 (PMC3446360; doi:10.1186/bcr3215)
Supplement: Additional file 2 — Supplementary Table 2. Real time (RT)-PCR primer sequences. [file bcr3215-S2.PDF]

**Table S2. RT-PCR Primer Sequences**

| Gene | Primer Sequence 5′ – 3′ |
|---|---|
| CD14 | ACATAAACTGTCAGAGGCAGCCGA<br>TAGCTTCTTTCCTACACAGCGGCA |
| CD68 | AACAAGCAATAGCACTGCCACCAG<br>TGTTGGATGAACCGTGGCATTTCC |
| AMAC1 | AGAGGAGTTGTGAGTTTCCAAGCC<br>AGGCAGCAGAGCTCTTTGTTGGTA |
| IL-10 | ATGCCCCAAGCTGAGAACCAAGACCCA<br>TCTCAAGGGGCTGGGTCAGCTATCCCA |
| TGFβ1 | ACACACTGCAAGTGGACATCAACG<br>TCTTCTCCGTGGAGCTGAAGCAAT |
| TNFα | ACCCTCAACCTCTTCTGGCTCAAA<br>AGGCCTAAGGTCCACTTGTGTCAA |
| IL-1β | AACAGGCTGCTCTGGGATTCTCTT<br>TGAAGGGAAAGAAGGTGCTCAGGT |
| β2M | GGCTGGCAACTTAGAG<br>GCCTTACTTTATCAAATGTAT |
| GAPDH | CCCTCCATTGACCTCAACTAC<br>CCACCTTCTTGATGTCATCAT |
