# Supplementary material for: Paracrine interactions between primary human macrophages and human fibroblasts enhance murine mammary gland humanization in vivo
Source: Breast Cancer Res. 2012 Jun 25;14(3):R97. doi: 10.1186/bcr3215 (PMC3446360; doi:10.1186/bcr3215)
Supplement: Additional file 3 — Supplementary Figure 1. Representative images of macrophages treated in culture and real time (RT)- PCR analysis of the activation stages of macrophages in vitro. [file bcr3215-S3.PDF]

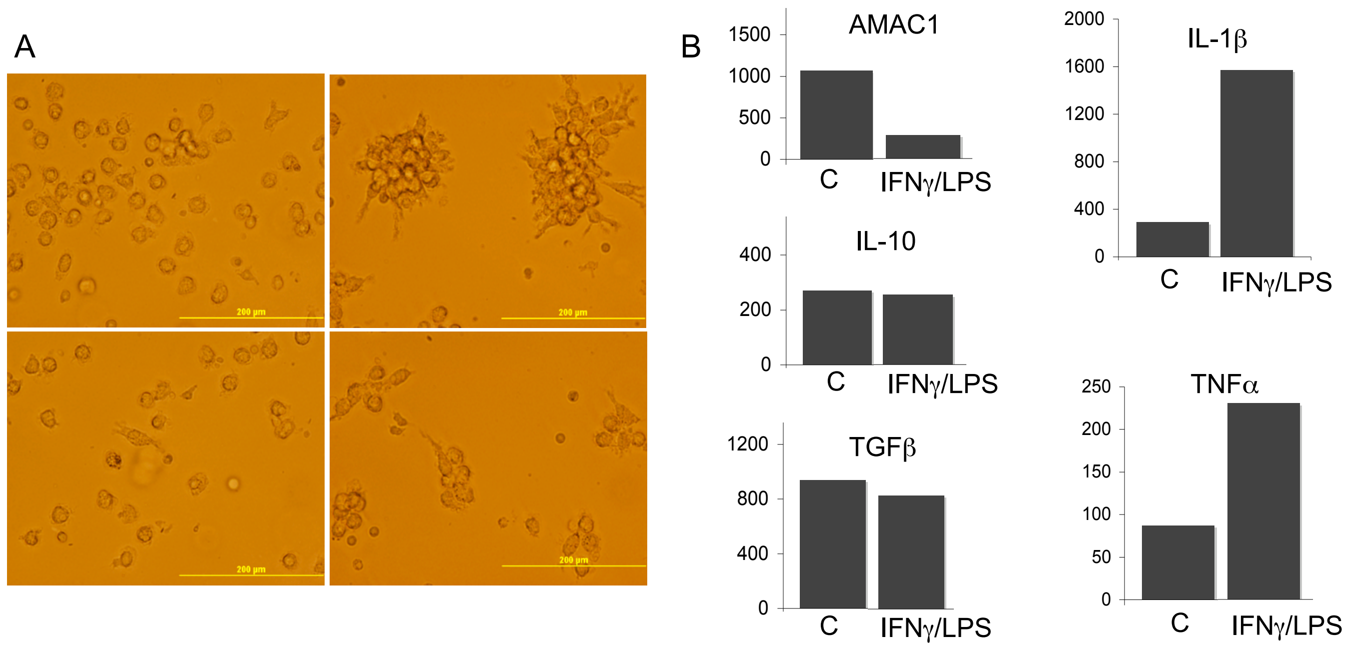

Figure S1. Activation stage of macrophages differentiated *in vitro*. Macrophages were isolated from apheresis products of premenopausal women via Ficoll separation and adherence to cell culture dishes, and then treated for three days with 20 ng/ml of human recombinant IFNγ and LPS. (A) Macrophages freshly isolated (left panels) and 3 days post-differentiation treatment (right panels): 20x magnification. (B) RT-PCR analysis of genes reported to be associated with alternatively or classically activated macrophages (left and right respectively). Data shown depicts one representative experiment of four independent experiments.
