# Supplementary material for: Paracrine interactions between primary human macrophages and human fibroblasts enhance murine mammary gland humanization in vivo
Source: Breast Cancer Res. 2012 Jun 25;14(3):R97. doi: 10.1186/bcr3215 (PMC3446360; doi:10.1186/bcr3215)
Supplement: Additional file 4 — Supplementary Figure 2. Graphs of macrophage stimulation of primary breast fibroblast proliferation in vitro. [file bcr3215-S4.PDF]

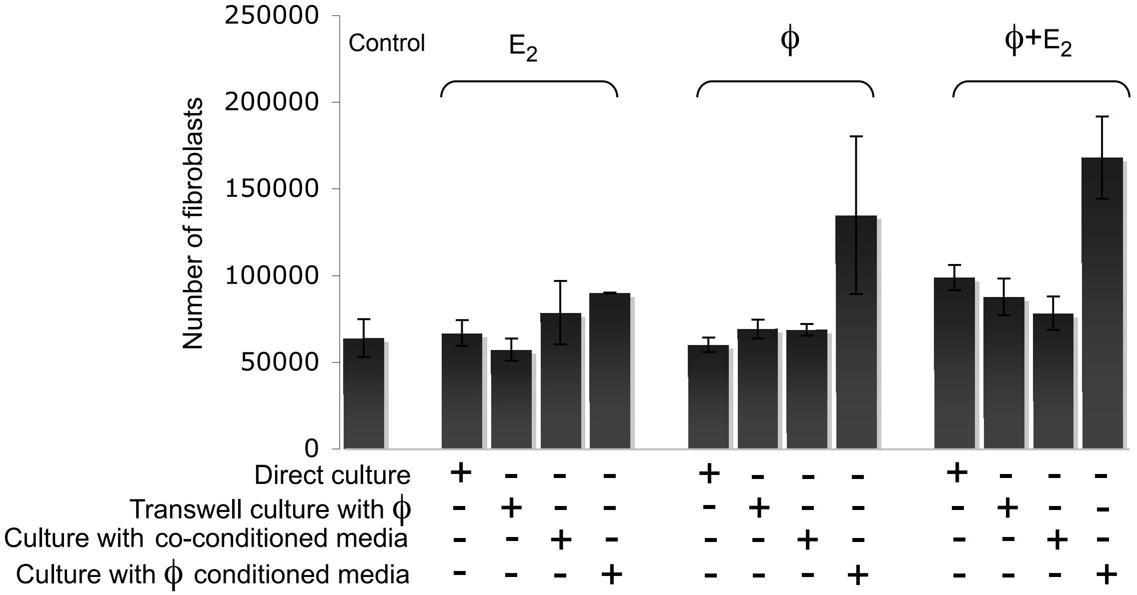


**Figure S2. Macrophages stimulate primary breast fibroblast proliferation**. Primary human monocytes/macrophages were isolated, differentiated, treated +/- estrogen ($10^{-10}$ M) for 24 hr and conditioned media were collected. (A) Primary human breast fibroblasts were placed in 2% charcoal stripped serum containing media 24 hr prior to treatment +/- estrogen or with macrophages +/- estrogen under the following conditions: (1) culturing directly with macrophages, (2) limiting exposure to macrophages by transwell membrane, (3) treating with co-conditioned media, (4) treating solely with macrophage conditioned media. For each assay, cells were treated for three days, then trypsinized and counted. Data shown are from one representative experiment of two independent experiments.
