# Supplementary material for: Paracrine interactions between primary human macrophages and human fibroblasts enhance murine mammary gland humanization in vivo
Source: Breast Cancer Res. 2012 Jun 25;14(3):R97. doi: 10.1186/bcr3215 (PMC3446360; doi:10.1186/bcr3215)
Supplement: Additional file 5 — Supplementary Figure 3. Ponceau staining of membranes, quantitation of zymography and western analysis, and representative images of ImageJ quantitation of stained tissue histosections. [file bcr3215-S5.PDF]

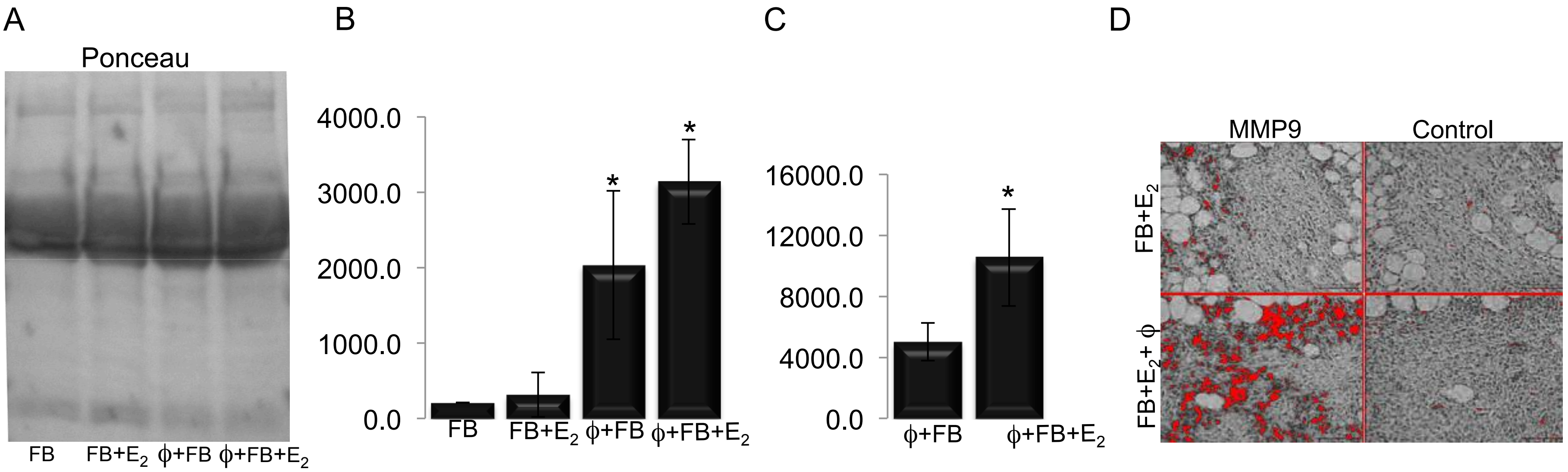

Figure S3. (A) Ponceau stained membrane displaying equally loaded protein in each lane. Results are representative of a four independent experiments. (B) Quantitation of gelatin zymography; results are mean +/- SD of four independent experiments. *P*<0.01. (C) Quantitation of western blot analyses; results are mean +/- SD of four independent experiments. **P*<0.02. (D) ImageJ64 measurement of threshold value, highlighting the expression of MMP9 in tissue histosections.
