# Supplementary material for: Paracrine interactions between primary human macrophages and human fibroblasts enhance murine mammary gland humanization in vivo
Source: Breast Cancer Res. 2012 Jun 25;14(3):R97. doi: 10.1186/bcr3215 (PMC3446360; doi:10.1186/bcr3215)
Supplement: Additional file 6 — Supplementary Figure 4. Graph comparing glands humanized with conditioned media vs. macrophages. [file bcr3215-S6.PDF]

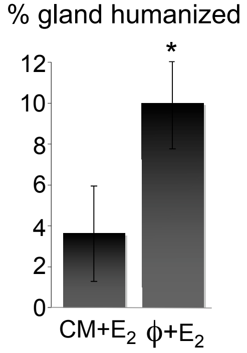

Figure S4. Concentrated macrophage conditioned media fails to recapitulate enhanced humanization. Primary human macrophages were treated +/- estrogen (10^{-10} M) in 2% charcoal stripped serum and 24 hr conditioned media were collected. Murine mammary glands were humanized with primary breast fibroblasts +/- concentrated macrophage conditioned media or primary macrophages. Glands were isolated two wk post-injection. A minimum of ten glands per treatment group were measured. *$P<0.05$. Total and humanized areas were determined as described in Figure 1.
