# Supplementary material for: Paracrine interactions between primary human macrophages and human fibroblasts enhance murine mammary gland humanization in vivo
Source: Breast Cancer Res. 2012 Jun 25;14(3):R97. doi: 10.1186/bcr3215 (PMC3446360; doi:10.1186/bcr3215)
Supplement: Additional file 7 — Supplementary Table 3. Table of values obtained from conditioned media ELISAs. [file bcr3215-S7.PDF]

Table S3. Estrogen treatment *and* fibroblast co-culture decrease cytokine/chemokines levels (pg/ml)

	IL-8	IL-23	Eotaxin
ϕ	19,304.5 ± 966.0	358.7 ± 57.9	2,072.2 ± 371.3
ϕ + E_2	1,751.4 ± 190.3	ND	282.9 ± 33.7
FB + E_2	6,168.9 ± 98.7	125.9 ± 60.3	458.4 ± 0.4
FB ϕ + E_2	1,319.7 ± 165.4	ND	148.6 ± 14.7

ϕ, macrophage-conditioned media; E_2, estrogen treatment; FB, fibroblasts-conditioned media; SD, standard deviation; ND, not detectable above basal media or estrogen containing media levels.
